# A Novel Class of *Schistosoma mansoni* Histone Deacetylase 8 (HDAC8) Inhibitors Identified by Structure-Based Virtual Screening and In Vitro Testing

**DOI:** 10.3390/molecules23030566

**Published:** 2018-03-02

**Authors:** Conrad V. Simoben, Dina Robaa, Alokta Chakrabarti, Karin Schmidtkunz, Martin Marek, Julien Lancelot, Srinivasaraghavan Kannan, Jelena Melesina, Tajith B. Shaik, Raymond J. Pierce, Christophe Romier, Manfred Jung, Wolfgang Sippl

**Affiliations:** 1 Department of Pharmaceutical Chemistry, University Halle-Wittenberg, 06120 Halle/Saale, Germany; veranso.conrad@gmail.com (C.V.S.); dina.robaa@pharmazie.uni-halle.de (D.R.); raghavk@bii.a-star.edu.sg (S.K.); jelena.melesina@pharmazie.uni-halle.de (J.M.); 2 Institute of Pharmaceutical Sciences, University of Freiburg, 79104 Freiburg, Germany; alokta.chakrabarti@gmail.com (A.C.); karin.schmidtkunz@pharmazie.uni-freiburg.de (K.S.); manfred.jung@pharmazie.uni-freiburg.de (M.J.); 3 Département de Biologie Structurale Intégrative, Institut de Génétique et Biologie Moléculaire et Cellulaire (IGBMC), Université de Strasbourg, CNRS, INSERM, B.P. 10142, 67404 Illkirch CEDEX, France; martin.marek@recetox.muni.cz (M.M.); shaik@igbmc.fr (T.B.S.); romier@igbmc.fr (C.R.); 4 Institut Pasteur de Lille, U1019—UMR 8204-CIIL-Centre d’Infection et d’Immunité de Lille, CNRS, Inserm, CHU Lille, Universite de Lille, F-59000 Lille, France; julien.lancelot@pasteur-lille.fr (J.L.); Raymond.Pierce@pasteur-lille.fr (R.J.P.)

**Keywords:** epigenetics, crystal structure, docking, histone deacetylase (HDAC) inhibitors, schistosomiasis, virtual screening

## Abstract

A promising means in the search of new small molecules for the treatment of schistosomiasis (amongst other parasitic ailments) is by targeting the parasitic epigenome. In the present study, a docking based virtual screening procedure using the crystal structure of histone deacetylase 8 from *Schistosoma mansoni* (smHDAC8) was designed. From the developed screening protocol, we were able to identify eight novel *N*-(2,5-dioxopyrrolidin-3-yl)-*n*-alkylhydroxamate derivatives as smHDAC8 inhibitors with IC_50_ values ranging from 4.4–20.3 µM against smHDAC8. These newly identified inhibitors were further tested against human histone deacetylases (hsHDAC1, 6 and 8), and were found also to be exerting interesting activity against them. In silico prediction of the docking pose of the compounds was confirmed by the resolved crystal structure of one of the identified hits. This confirmed these compounds were able to chelate the catalytic zinc ion in a bidentate fashion, whilst showing an inverted binding mode of the hydroxamate group when compared to the reported smHDAC8/hydroxamates crystal structures. Therefore, they can be considered as new potential scaffold for the development of new smHDAC8 inhibitors by further investigation of their structure–activity relationship.

## 1. Introduction

Schistosomiasis, one of the most important neglected tropical diseases, is endemic in 75 countries worldwide, especially in Africa, Asia, South America and the Middle East [1,2]. The disease is caused by trematodes of the genus *Schistosoma*, with *S. mansoni, S. japonicum* and *S. haematobium* being the main causative parasites of human schistosomiasis. According to the World Health Organisation (WHO), over 240 million people are infected and more than 200 million people require preventive treatment yearly [3]. Chronic schistosomiasis is associated with serious morbidity and may cause long-term irreversible consequences such as liver fibrosis, kidney failure, as well as bladder and cervical cancer and other malignancies [4,5]. Praziquantel was introduced in the mid-1970s, and remains practically the only used drug for the treatment and control of schistosomiasis [5,6]. The exact mechanism of its antiparasitic activity, however, is poorly understood [6,7]. Praziquantel is a low-cost and highly effective drug, which is active against all *Schistosoma* species and is orally administered as a single dose, showing no notable side effects [4,6,7]. However, a major drawback is the lack of efficacy against immature parasites, in some cases leading to treatment failure [7,8]. Serious concerns have been raised over the potential for emergence of praziquantel resistance, especially because of its long-term use as a sole drug, both in the treatment and prevention of schistosomal infections, as well as its implementation in mass drug administration campaigns [7,8,9,10,11]. Several reports describe incidences of reduced efficacy of praziquantel against some *Schistosoma* species as well as the induction of drug resistance in laboratory strains [12,13,14,15,16,17,18]. This emphasizes the urgent need to develop novel and alternative antischistosomal agents.

In recent years, targeting the parasitic epigenome has emerged as a new and promising strategy to tackle several parasites such as *Schistosoma*, *Toxoplasma*, *Plasmodium*, *Trypanosoma* and *Leishmania* species [19,20]. In this regard, Zn-dependent histone deacetylases (HDACs) have emerged as highly attractive targets, especially since they are well-recognized as validated targets in cancer therapy. Indeed, several studies have demonstrated the role of HDACs in the life cycle of *Schistosoma*; class I HDACs (smHDAC1, 3, and 8) were found to be expressed in all stages of *Schistosoma* lifecycle, with smHDAC8 showing the highest abundance [21]. Treatment of the parasites with pan-HDAC inhibitors was found to induce schistosomes mortality [22,23]. However, with the objective of developing candidate drugs against schistosomiasis and to limit potential side-effects, it is advisable to target individual schistosome HDACs. We showed that mice infected with schistosomula knocked down for smHDAC8 transcripts showed a decreased number of recovered adult worms and lower egg burden [24], suggesting that this enzyme is a valid therapeutic target. Notably, the human orthologue of smHDAC8, hsHDAC8, generally shows less abundance in humans than other class I HDACs (HDAC1 and 3) and is only upregulated in some tumor cells [25]. Therefore, small-molecule smHDAC8 inhibitors represented a promising approach for the treatment of schistosomiasis.

The majority of reported HDAC inhibitors (HDACi) possess a common pharmacophore entailing a “warhead”, which is a functional group that is able to chelate the catalytic zinc ion, a linker region, embedded in the hydrophobic lysine tunnel, and a “cap group” that interacts with the residues on the rim of the substrate binding pocket and which, in some cases, can impart subtype selectivity of the compounds. The vast majority of HDACi possess a hydroxamate group as a warhead, since it is able to strongly chelate the zinc ion [26]. Crystal structures of various HDACs with hydroxamate derivatives show that, in most cases, the hydroxamate group chelates the catalytic zinc ion in a bidentate fashion and is further stabilized by undergoing a hydrogen bond triad with the two conserved histidine residues and the catalytic tyrosine residue in the catalytic pocket [27]. Nevertheless, several structures also show hydroxamate derivatives that only coordinate the zinc ion in a monodentate fashion, as clearly seen in some of the newly released crystal structures of zebrafish HDAC6 [28,29]. Alternative Zn-chelating groups found in reported HDACi include azetidinone, cyclic thiourea, thiol, carboxylic acid, amino acid, and *ortho*-aminoanilide groups [30,31]. Importantly, HDACi containing *ortho*-aminoanilides tend to show preferential inhibition of class I HDACs [31], while those encompassing aryl-amino acid cores showed selectivity for HDAC8 over other HDAC subtypes [32]. The selectivity of *ortho*-aminoanilides and amino acid derivatives was found to arise from the exploitation of the acetate release channel, also known as the “foot pocket” [32,33].

In our previous studies, we have reported on the virtual screening-based identification and structure-guided optimization of arylhydroxamate derivatives as smHDAC8 inhibitors [24,34,35]. Several of the optimized benzamidohydroxamates were found to exhibit high inhibitory potency for smHDAC8, selectivity over relevant human HDAC isoforms (HDAC1, 3 and 6; in some cases also HDAC8), and to induce apoptosis and mortality of schistosomes in cellular assays [35].

In order to identify other lead scaffolds as smHDAC8 inhibitors, we performed a new structure-based virtual screening and tested some selected hits using validated enzyme inhibition assays. The smHDAC8 crystal structure was used to virtually screen the Interbioscreen compound library comprising of about 550,000 molecules. Compounds that were successfully docked and showed favourable interaction with the catalytic zinc ion and the smHDAC8 binding pocket were selected. We further excluded all hydroxamate derivatives with the motif –C=O-NH-OH, since we have already exploited this class of inhibitors in our previous studies, and focused on compounds showing alternative groups for the in vitro experiments.

## 2. Results and Discussion

### 2.1. Novel smHDAC8 Inhibitor(s) Identified from the Virtual Screening

**Figure 1:**
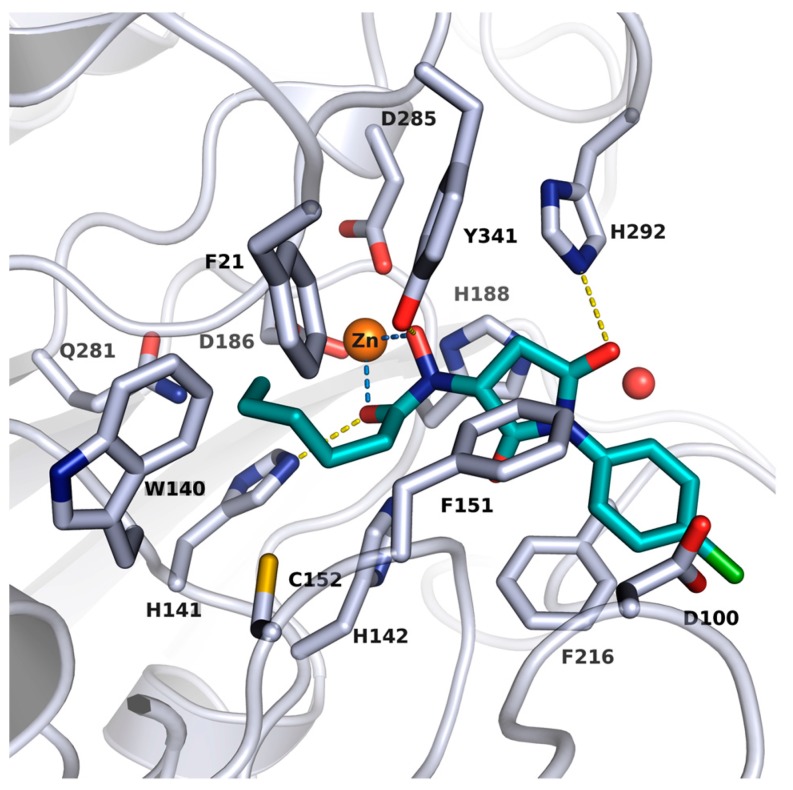
Docking pose of J1036 in smHDAC8 (PDB ID: 4BZ8). Protein backbone is shown as a ribbon and side chains of key amino acid residues in the active site are shown as white sticks. The catalytic zinc ion and water molecule are shown as an orange and red sphere, respectively. Coordination of the zinc ion by J1036 is represented with light blue lines while the hydrogen bond interaction between the docked hit and the protein is shown as a yellow line.

In order to identify novel inhibitors for smHDAC8, we applied a virtual database screening strategy using the Interbioscreen database comprising of about 550,000 compounds. The reported crystal structure of smHDAC8 (PDB code 4BZ8) in complex with J1038 (1,4-benzothiazine-6-hydroxamate), was used for the virtual screening study utilizing GLIDE docking program (version 2017-2). By using this setup, the cocrystallized ligand could be correctly redocked into the binding pocket of smHDAC8 (rmsd below 0.5 Å). Our search was focused on compounds with chemical moieties that are structurally related to the hydroxamate group as zinc chelator. To this end, a substructure search for similar substances was conducted in the Interbioscreen database. The majority of the retrieved compounds (828 structures) were *N*-oxidized urea derivatives, which were subsequently discarded since previous studies have shown that compounds bearing this moiety as a zinc binding warhead are less effective as HDAC inhibitors than their hydroxamic acid counterparts [36]. Eighty compounds (Table S1, Supplementary Material) were finally retained and docked into the smHDAC8 binding pocket. The final selection of a smaller subset of compounds was based on the derived docking scores, pan-assay interference compounds (PAINS) filtering, molecular weight (fragments with MW < 250 were not considered for testing, since they are usually weakly active and difficult to measure using standard in vitro assays), and a visual inspection of the predicted binding mode (see Methods section for details). Particularly interesting was a series of *N*-(2,5-dioxopyrrolidin-3-yl)hydroxamate derivatives bearing a reverse *n*-alkyl substituted hydroxamate group—a scaffold not been previously reported as an HDACi [37,38]. Our docking studies revealed that the substituted hydroxamate group binds in an inverted manner when compared to the reported HDACs/hydroxamates crystal structures, but is still able to chelate the catalytic zinc ion in a bidentate fashion. It was also remarkable from the docking poses of these compounds that the *n*-alkyl group can be accommodated in the acetate-binding cavity (upper region of the foot pocket) of smHDAC8, where it can undergo vdW contacts with the surrounding aromatic acid residues. Meanwhile, the pyrrolidinedione moiety of the ligands can undergo a hydrogen bond interaction with the side chain of His292 and additionally the phenyl capping group extends into the side pocket showing π–π stacking interaction with Phe216 (Figure 1). Hence, nine *N*-(2,5-dioxopyrrolidin-3-yl)-*n*-alkylhydroxamate derivatives were selected, purchased from Interbioscreen, and submitted to an in vitro assay to test their inhibitory potency against smHDAC8 (Table 1). The selected compounds bear *n*-alkyl groups of different lengths, ranging from *n*-butyl to *n*-hexyl moieties, and feature different substituents at the *N*-phenyl group.

The biological assay was carried out using purified smHDAC8 protein and the small molecule substrate Z(Tfa)Lys-AMC (ZMTFAL) as previously reported [39,40]. To assess the selectivity, the selected compounds were further tested against the human orthologue hsHDAC8 as well as the major human HDAC isoforms (HDAC1 and -6) using an established in vitro assay [34]. Of the nine selected virtual screening hits, eight compounds showed an inhibitory activity against smHDAC8 with IC_50_ values in the low micromolar range (IC_50_ 4.4–20.3 µM). J1036, bearing an *n*-pentylhydroxamate group and an *N*-(4-chlorophenyl) substituent, showed the highest inhibitory potency against smHDAC8 among all tested compounds. All the tested compounds were also active against the human orthologue, mostly showing an increased inhibitory activity towards hsHDAC8 as compared to smHDAC8. Moreover, the compounds were found to inhibit HDAC1 and HDAC6 isoforms, with three compounds (J1060, J1063 and J1064) showing a submicromolar inhibitory activity against HDAC6.

### 2.2. X-ray Structure of smHDAC8 in Complex with J1036

**Figure 2:**
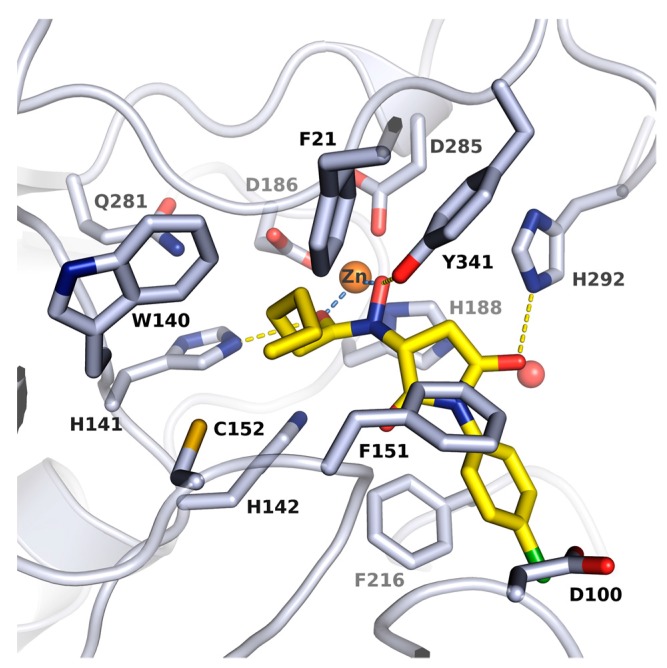
Crystal structure of smHDAC8/J1036 complex. Close-up view of the binding mode of J1036 in the smHDAC8 active-site cleft. Protein residues are shown as grey sticks, and J1036 is shown as yellow sticks. The catalytic zinc is shown as orange sphere while conserved water molecule is shown as a red sphere. Coordinations to zinc ion and hydrogen bond interactions to the protein are shown as dashed light blue and yellow lines respectively.

To get molecular insight into the mode of action of the newly identified inhibitors bearing *n*-alkyl substituted hydroxamate moiety, the crystallographic structure of the complex between smHDAC8 and J1036 compound at 1.55 Å was determined and refined (Table S2, Supplementary Material). The electron density for the inhibitor was not perfect but its binding at the catalytic zinc could be unambiguously assessed. Specifically, the inhibitor structure is observed in monomer A (as shown in Figure 2). We observed only part of the inhibitor structure in monomer B. Here, the hydroxamate is interacting with the zinc ion and the long alky chain is located in the foot pocket, indicating that the compound is partially hydrolyzed under the X-ray conditions. This is confirmed by the observed binding of the cleaved capping group of J1036 in a noncatalytic smHDAC8 pocket. Further experiments with novel analogs have to be carried out to analyze this effect in more detail.

Analysis of the crystal structure of the smHDAC8/J1036 complex in monomer A shows that the inhibitor binds in the smHDAC8 active-site pocket, where it forms specific interactions with the enzyme (Figure S1, Supplementary Material). As shown in Figure 2, the internal hydroxamate group is coordinating smHDAC8 catalytic zinc ion in a bidentate fashion. The n-pentyl-hydroxamate group additionally interacts via hydrogen bonding with three residues, namely His141, His142 and Tyr341. The last of these residues adopts the flipped-in conformation that is typically seen in all HDAC/hydroxamate complexes. The J1036 n-pentyl moiety is deeply buried in the smHDAC8 foot-pocket, underneath the catalytic zinc ion, where it makes non-polar contacts with smHDAC8 residues Phe21, Trp140 and Cys152. The J1036 pyrrolidinedione linker moiety further forms a hydrogen bond interaction with smHDAC8 His292 side chain. Finally, the halogen-substituted phenyl capping group of J1036 forms parallel-displaced π–π stacking with Phe216.

### 2.3. Docking into X-ray Structures of Human HDACs

**Figure 3:**
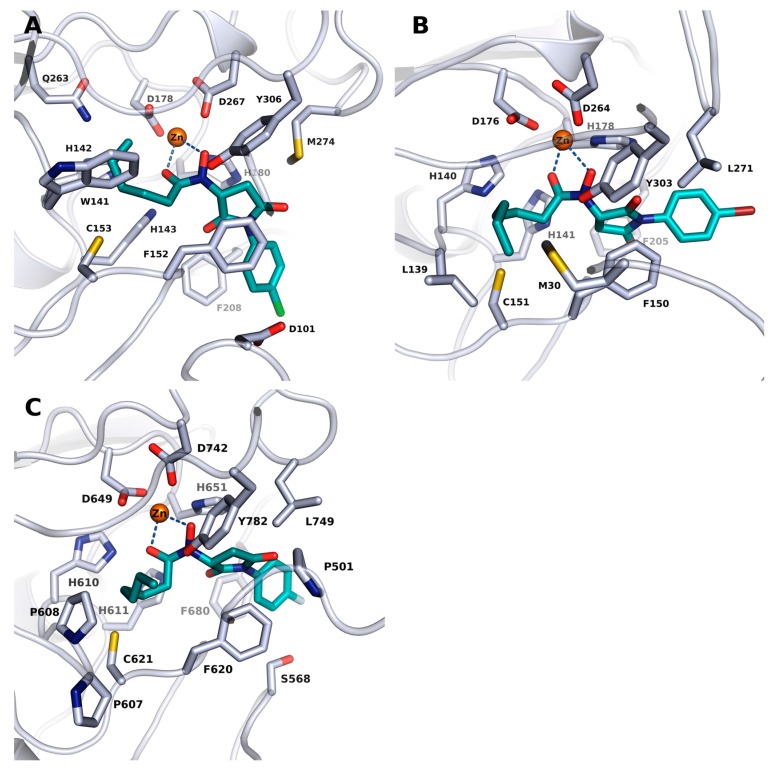
Docking poses of J1036, J1063 and J1064 in the human HDAC8 (**A**), HDAC1 (**B**) and CD2 HDAC6 (**C**) isoforms, respectively. Ligands are shown in cyan while the protein backbones are shown as ribbons and side chains of key amino acid residues in their respective active sites are shown as white sticks. Catalytic zinc ion is shown as an orange sphere. Coordination of the zinc ion is represented with light blue lines.

In an attempt to explain the observed inhibitory activity of the identified compounds against the herein tested human HDAC isoforms, docking studies were carried out using available HDAC8 (PDB ID: 2V5X), HDAC6 (PDB ID: 5EDU) and HDAC1 (PDB ID: 5ICN) crystal structures. The docking protocol was first validated for the selected HDACs by redocking the cocrystallized ligands into the catalytic pocket of each protein–ligand complex. Generally, the binding pose of the cocrystallized ligands could be reproduced by the docking program with rmsd values <1.5 Å with their respective crystal structure. As could be expected, the zinc chelating hydroxamate group of the ligands, as exemplified by J1036, demonstrated the most potent activity against hsHDAC8 (Figure 3A). J1036 was oriented in the active site of hsHDAC8 in a similar way to the observed binding mode of the resolved smHDAC8/J1036 crystal structure (see Section 2.2) and the *n*-alkyl group was accommodated into the acetate-release channel. From the observed docking poses, a bidentate coordination of the zinc ion was possible via the hydroxamate carbonyl and hydroxyl moiety, and the phenyl capping group could be embedded into the side pocket forming additional π–π stacking interactions. A similar binding mode could be predicted by our docking studies for the identified hits in HDAC1 as demonstrated by J1063 which had the best activity against this isoform (Figure 3B).

Docking into the available crystal structure of hsHDAC6, however, yielded in a first-run no favourable binding mode, which could explain the high activity of the compounds against HDAC6. In order to explain this observed activity, we exploited the active site of human HDAC6 (PDB ID: 5EDU) in an attempt to explore possible conformations of the acetate-binding pocket, such that larger molecules could fit. This was done by inserting the J1036 molecule from the resolved smHDAC8 crystal structure into the hsHDAC6 crystal structure and subsequently minimizing the protein structure. This minimization step resulted only in a slight conformational change of the side chain residues of the acetate binding pocket (Figure S2, Supplementary Material). Docking of the identified hits into this modified hsHDAC6 structure yielded similar binding modes as those observed for the other HDAC isoforms, where the hydroxamate groups show a bidentate chelation to the catalytic zinc ion and the *n*-alkyl group occupies the acetate-binding cavity as exemplified by J1064, which showed the highest activity against hsHDAC6 (Figure 3C).

### 2.4. The n-Alkylhydroxamate Derivative J1036 Induces Apoptosis in S. mansoni Larvae

**Figure 4:**
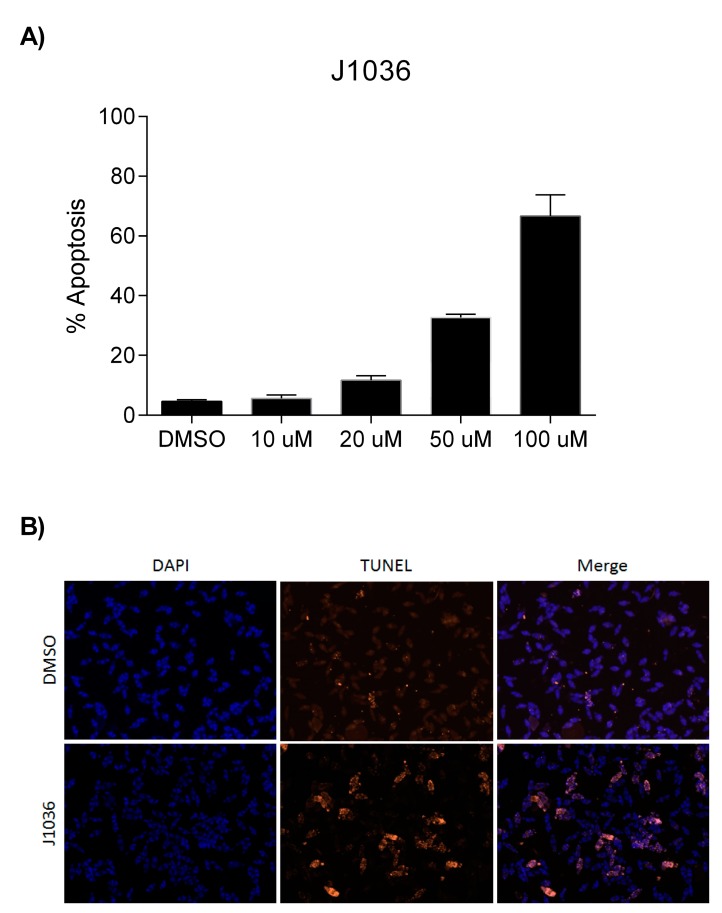
J1036 induces apoptosis in *S. mansoni* schistosomula in vitro. (**A**) Dose-dependent induction of apoptosis determined by dUTP nick end labeling (TUNEL) shown as the percentage of parasites positively labeled; (**B**) TUNEL staining of schistosomula treated with 100 µM J1036 for 3 days. Parasites were counterstained using 4′,6-Diamidino-2-Phenylindole (DAPI).

The compound J1036 induced dose-dependent apoptosis in the infective larval stage (schistosomula) of *S. mansoni*, affecting 67% of the larvae after 3 days at a dose of 100 µM (Figure 4), whereas a polar hydroxamate derivative (J1038) identified by a previous virtual screening [24] was ineffective in the same assay (data not shown here). This is comparable to the effect achieved with the pan-HDAC inhibitor SAHA (43% at 100 µM) and the previously-described linker-less, lipophilic hydroxamate derivative J1075 (54% at 100 µM) [24]. It is, however, less effective than the benzamidohydroxamates we also developed [35], which were also more efficient smHDAC8 inhibitors, some with low nM inhibitory IC_50_ values. Although we cannot directly correlate the inhibitory activity on smHDAC8 with the lethal effect of J1036 on schistosomula, the high level of expression of this class I HDAC [21] in the parasite, and its validation as a stand-alone target by transcript knockdown [24], mean that its inhibition is sufficient to induce the effects observed.

## 3. Materials and Methods 

### 3.1. Computational Methods

#### 3.1.1. Molecular Docking

The ligands and protein–ligand complexes used herein were prepared using a similar method as reported in our previous published paper [35].

##### Ligand Preparation

The ligands were prepared for docking using the LigPrep tool [41] as implemented in Schrödinger’s software (version 2017-2), where all possible tautomeric forms, as well as stereoisomers, were generated. They were subsequently energy minimized using the integrated Optimized Potentials for Liquid Simulations (OPLS_2005) force field [42]. 25 conformers of prepared ligands were calculated with ConfGen using the default settings and allowing minimization of the output conformations [43,44].

##### Protein Preparation

The crystal structures of HDAC8 (hsHDAC8; PDB ID: 2V5X), smHDAC8 (PDB ID: 4BZ8), HDAC6 (PDB ID: 5EDU) and HDAC1 (PDB ID: 5ICN) were downloaded from the Protein Databank (PDB; www.rcsb.org) [45]. With the exception of water molecules occupying the catalytic pockets that were used for the docking procedures, all water molecules were deleted using the MOE software [46]. Further preparations of the protein structures were done using the Protein Preparation Wizard of Schrödinger software [47,48]. Bond orders were assigned and hydrogen atoms added, and the H-bond network was subsequently optimized. The protonation states at pH 7.0 were predicted using the Epik-tool in Schrödinger [49,50]. The structures were finally subjected to a restrained energy minimization step (rmsd of the atom displacement for terminating the minimization was 0.3 Å) using the OPLS2005 force field [42].

HDAC6 (PDB ID: 5EDU) was specially treated as follows: J1036 was retrieved from the herein described crystal structure of smHDAC8 and used in place of the cocrystallized ligand of the 5EDU structure during the protein preparation and minimization steps as mentioned above. A further docking procedure in HDAC6 was then conducted using the minimized structure.

##### Virtual Screening

A docking protocol using the Glide program [51] was developed and validated by redocking the cocrystallized inhibitors (benzhydroxamates) of smHDAC8 (PDB ID: 4BZ8, 5FUE) with the corresponding crystal structure. Only the top-ranked docking pose for each ligand were kept and subsequently subjected to binding free energy calculation using the Amber12EHT force field and the GBSA solvation model implemented in MOE. For screening the Interbioscreen (https://www.ibscreen.com/database) comprising of about 550,000 compounds was used. First, a substructure search was conducted in the Interbioscreen database. The majority of the retrieved compounds (828 structures) were *N*-oxidized urea derivatives, which were subsequently discarded. 80 compounds (Data see Table S1, Supplementary Material) were finally retained and docked into the smHDAC8 binding pocket. Compounds with the motif –C=O-NH-OH (classical hydroxamates), were not considered since we have already exploited this class of inhibitors in our previous studies. In addition, fragments with MW < 250 da and compounds predicted to be pan-assay interference compounds (PAINS) were rejected [52]. Filtering the database for PAINS was performed using PAINS1, PAINS2, and PAINS3 filters as implemented in Schrödinger’s Canvas program [53]. A total of nine compounds were finally purchased from Interbioscreen (Table 1).

##### Docking to Human HDACs

Docking procedures were done using the Glide program [51,54,55]. The receptor grid preparation for the docking procedure was carried out by assigning the co-crystallized ligand as the centroid of the grid box with an additional zinc metal constraint. The generated 3D conformers (refer to Section 3.1.1) were docked into the receptor model with zinc metal constraint using Glide [51,54,55] and the Standard Precision (SP) mode as the scoring function. A total of 60 poses per ligand conformer were included in the post-docking minimization step, and a maximum of 20 docking poses was generated for each ligand conformer.

### 3.2. Activity and Inhibition Assays

Recombinant human HDAC1 and HDAC6 were purchased from BPS Bioscience. Recombinant human HDAC8 was expressed and purified as previously described [24]. Recombinant smHDAC8 enzyme was produced in *E. coli* cells and purified by a method previously described by us [24]. Inhibition assays of smHDAC8 and human HDACs were performed as already described in previous publications [24,56]. Briefly, the commercial Fluor de Lys kit (BML-KI178) was used for testing inhibition of smHDAC8 and hsHDAC8. Test compounds, Fluor de Lys-HDAC8 substrate (50 µM) and enzyme were incubated for 90 min at 37 °C with subsequent addition of 50 μL Developer II (BML-KI176) and further incubation for 45 min at 30 °C. Fluorescence was measured in a plate reader (BMG Polarstar) with excitation at λ = 390 nm and emission at λ = 460 nm. Inhibition tests of human HDAC1 and 6 were conducted using ZMAL (Cbz-(Ac)Lys-AMC) as substrate and trypsin as a developer. After incubation of the compounds, ZMAL (10.5 µM) and enzyme for 90 min at 37 °C, 60 μL of trypsin was added and further incubated for 20 min at 37 °C. Trichostatin A (2 µM) was used in both assays to stop the reaction. Fluorescence was measured similarly as mentioned above. IC_50_ values were determined with OriginPro (version 9.0.0, Northampton, MA, USA). IC_50_ Values in Table 1 are given as mean ± S.E.

### 3.3. Phenotypic Screening

The capacity of compounds J1036 and J1038 to induce apoptosis in *S. mansoni* schistosomula was measured using dUTP nick end labelling (TUNEL) and the In Situ Cell Death Detection Kit TMR (Roche Applied Science, Penzberg, Germany) exactly as previously described [23,24].

### 3.4. Crystallization and X-ray Structure Determination

The smHDAC8 enzyme was recombinantly produced as described previously [24]. Briefly, diffraction-quality crystals of native smHDAC8 enzyme were obtained at 17 °C after 3 days by mixing equal volumes of smHDAC8 (2.5 mg/mL) with reservoir solution composed of 21% PEG 3350 and 0.2 M Na^+^/K^+^ l-tartrate. After 3 days, grown crystals were soaked in mother liquor supplemented with the inhibitor J1036 (10 mM final concentration) for 20 h. Crystals used for crystallographic data collection were briefly transferred in a reservoir solution supplemented with 22% glycerol and flash-frozen in liquid nitrogen. Crystallographic data obtained in this project were collected at 100 K on SOLEIL beamline PROXIMA1. The crystallographic data were processed and scaled using HKL2000 [57], and the smHDAC8/J1036 complex structure was solved by rigid-body refinement using Phenix [58]. The initial model was refined through several cycles of manual building using Coot [59] and automated refinement with Phenix. The final model was validated using tools provided in Coot (see Table S2, Supplementary Material). Atomic coordinates and structure factors of the smHDAC8/J1036 complex were deposited in the Protein Data Bank under PDB ID 6FU1.

## 4. Conclusions

SmHDACs, and in particular smHDAC8, warrant attention as they are validated targets and are of potentially high therapeutic value in the search of new small molecules for the treatment of schistosomiasis. Virtual screening applications have been used as a rapid and economic strategy in lead discovery. In this study, we have employed a docking based virtual screening procedure to identify eight novel *N*-(2,5-dioxopyrrolidin-3-yl)-*n*-alkylhydroxamate derivatives. They have been found to be active in the low micromolar range (IC_50_ 4.4–20.3 µM) against smHDAC8 utilizing an established in vitro assay. The identified hits also showed interesting activities against the hsHDACs. Just like the well-known hydroxamates, the newly reported compounds described herein also coordinated the zinc ion in a bidentate fashion. Interestingly, the hydroxamate moiety binds in an inverted manner to the catalytic pocket when compared to all the so far reported crystal structures of HDACs/hydroxamate complexes. Detailed analyses of the binding mode using docking studies of the identified hits show that besides the chelation of the zinc ion, stabilization in the active site is also enhanced by the establishment of multiple hydrogen bonds with polar residues and vdW contacts with hydrophobic residues in the acetate binding pocket. Furthermore, the *n*-pentyl moiety of J1036 is placed in the foot pocket, which has not been observed for previously reported monosubstituted hydroxamates. The occupancy of the foot pocket offers the opportunity to develop selective HDAC inhibitors by further optimization as reported previously [32,33]. Another advantage of the newly discovered inhibitors is their expected lower toxicity in comparison to classical hydroxamates. The mutagenicity of hydroxamates is caused by formation of the isocyanate by Lossen rearrangement [31]. This toxicity mechanism might be absent for J1036 and its analogs due to substitution on the nitrogen atom of the hydroxamate moiety. Together with the effectiveness of J1036 in inducing apoptosis in the parasite, this means these lead molecules represent a useful starting point for designing new smHDAC8 inhibitors, as well as inhibitors of human HDAC isoforms.

## Figures and Tables

**Table 1:** In vitro inhibition of *N*-(2,5-dioxopyrrolidin-3-yl)-*n*-alkylhydroxamate against smHDAC8 and hsHDACs. SAHA and TH65 [35] were included as positive controls.

Cmpd.	IBS Code	Structure	smHDAC8 IC_50_ (µM)	hsHDAC8 IC_50_ (µM)	hsHDAC1 IC_50_ (µM)	hsHDAC6 IC_50_ (µM)
J1036	STOCK4S-53643	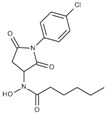	4.40 ± 0.17	0.49 ± 0.18	6.76 ± 0.97	5.02 ± 0.31
J1057	STOCK4S-48892	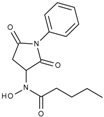	13.18 ± 1.85	2.62 ± 0.19	42.1 ± 2.20	6.20 ± 0.34
J1058	STOCK4S-78560	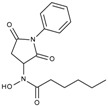	20.30 ± 2.78	3.99 ± 0.74	25.96 ± 2.40	6.20 ± 0.41
J1060	STOCK4S-27444	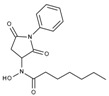	11.47 ± 0.91	1.80 ± 0.24	5.00 ± 0.42	0.86 ± 0.12
J1061	STOCK4S-02282	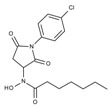	5.5 ± 0.7	7.69 ± 3.30	3.98 ± 0.45	2.65 ± 0.29
J1063	STOCK4S-11661	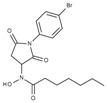	5.9 ± 1.6	7.72 ± 4.42	1.42 ± 0.13	0.77 ± 0.09
J1064	STOCK4S-11028	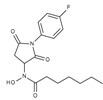	7.79 ± 0.28	2.08 ± 0.34	4.30 ± 0.46	0.60 ± 0.12
J1065	STOCK4S-31959	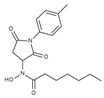	20.2 ± 2.7	3.96 ± 0.60	8.40 ± 0.28	1.57 ± 0.37
J1066	STOCK5S-25749	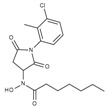	13% inhib. at 25 µM	n.d.	n.d.	n.d.
TH65		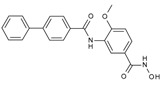	0.075 ± 0.025	0.026 ± 0.017	6.3 ± 2.1	0.390 ± 0.002
SAHA		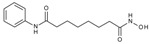	1.56 ± 0.20	0.40 ± 0.10	0.12 ± 0.01	0.104 ± 0.009

## Supplementary Materials

The following are available online. Figure S1: X-ray structure of smHDAC8/J1036 complex, Figure S2.: Superimposition of the CD2 of HDAC6 after minimization with J1036, Table S1: Pre-filtered Interbioscreen database (80 compounds) considered for the docking study, Table S2: smHDAC8/inhibitor X-ray structure. Data collection and refinement statistics.
