# Supplementary material for: A Novel Class of Schistosoma mansoni Histone Deacetylase 8 (HDAC8) Inhibitors Identified by Structure-Based Virtual Screening and In Vitro Testing
Source: Molecules. 2018 Mar 2;23(3):566. doi: 10.3390/molecules23030566 (PMC6017931; doi:10.3390/molecules23030566)
Supplement: Supplementary file 1 [file molecules-23-00566-s001.pdf]

# Supplementary material

# A novel class of *Schistosoma mansoni* histone deacetylase 8 (HDAC8) inhibitors identified by structure-based virtual screening and in vitro testing

**Conrad V. Simoben [1], Dina Robaa [1], Alokta Chakrabarti [2,†], Karin Schmidtkunz [2], Martin Marek [3,‡], Julien Lancelot [4], Srinivasaraghavan Kannan [1,§], Jelena Melesina [1], Tajith B. Shaik [3], Raymond J. Pierce [4], Christophe Romier [3], Manfred Jung [2] and Wolfgang Sippl [1,*]**

[1] Department of Pharmaceutical Chemistry, University Halle-Wittenberg, 06120 Halle/Saale, Germany; veranso.conrad@gmail.com (C.V.S.); dina.robaa@pharmazie.uni-halle.de (D.R.); raghavk@bii.a-star.edu.sg (S.K.); jelena.melesina@pharmazie.uni-halle.de (J.M.)

[2] Institute of Pharmaceutical Sciences, University of Freiburg, 79104 Freiburg, Germany; alokta.chakrabarti@gmail.com (A.C.); karin.schmidtkunz@pharmazie.uni-freiburg.de (K.S.); manfred.jung@pharmazie.uni-freiburg.de (M.J.)

[3] Département de Biologie Structurale Intégrative, Institut de Génétique et Biologie Moléculaire et Cellulaire (IGBMC), Université de Strasbourg, CNRS, INSERM, B.P. 10142, 67404 Illkirch Cedex, France; martin.marek@recetox.muni.cz (M.M.); shaik@igbmc.fr (T.B.S.); romier@igbmc.fr (C.R.)

[4] Institut Pasteur de Lille, U1019 - UMR 8204-CIIL-Centre d'Infection et d'Immunité de Lille, CNRS, Inserm, CHU Lille, Universite de Lille, F-59000 Lille, France; julien.lancelot@pasteur-lille.fr (J.L.); Raymond.Pierce@pasteur-lille.fr (R.J.P.)

* Correspondence: wolfgang.sippl@pharmazie.uni-halle.de; Tel.: +49 345 5525040

† Current address: ProQinase GmbH, 79106 Freiburg, Germany

‡ Current address: Loschmidt Laboratories, Department of Experimental Biology & Recetox, Masaryk University, 625 00 Brno, Czech Republic

§ Current address: Bioinformatics Institute (A*STAR), #07-01 Matrix, 138671 Singapore, Singapore

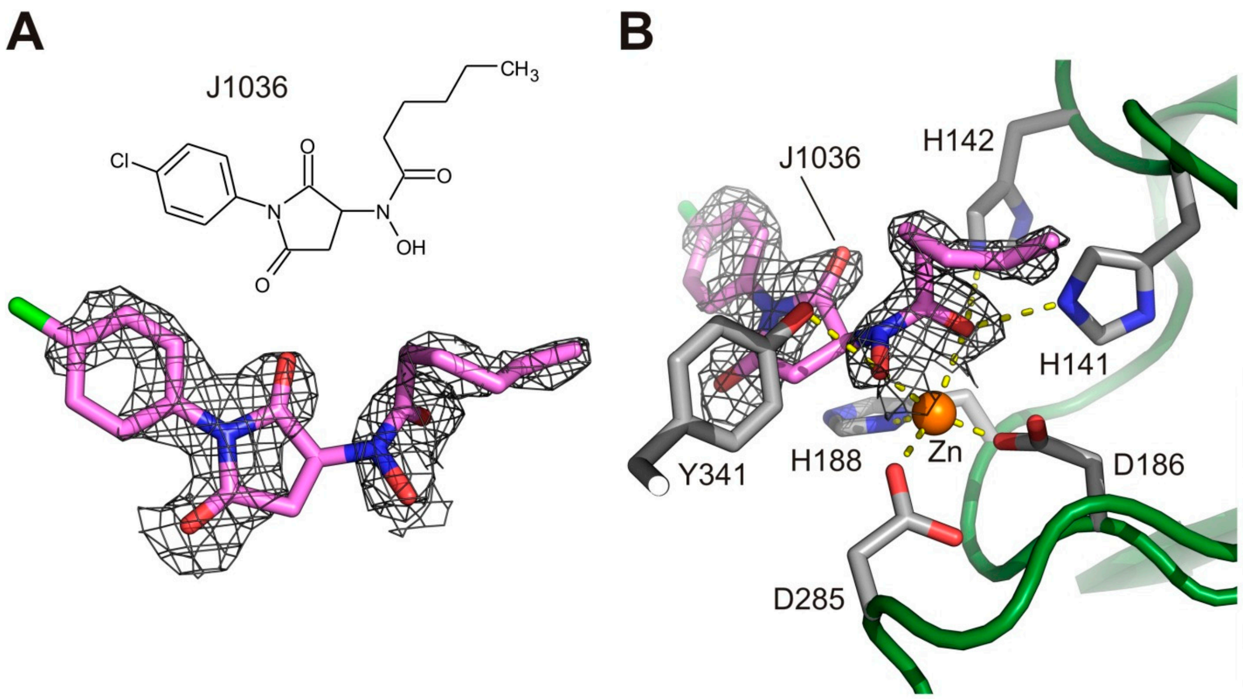


**Figure S1**. X-ray structure of smHDAC8/J1036 complex. A) Observed electron density (contour level 1σ) of the bound J1036 molecule B) smHDAC8/J1036 complex with electron density of bound J1036 (contour level 1σ). The inhibitor molecule is colored magenta, the zinc ion is colored gold.

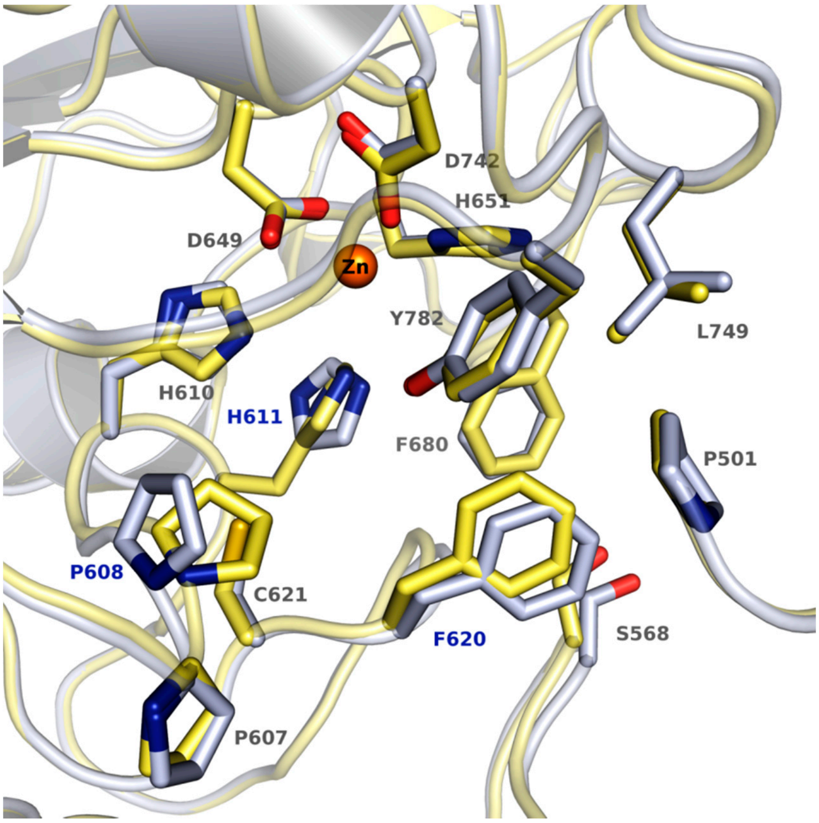


**Figure S2.** Superimposition of the CD2 of hsHDAC6 after minimization with J1036. Backbone of the original protein crystal structure (PDB ID: 5EDU) with closed pocket is shown as a yellow ribbon meanwhile the backbone of the open pocket structure after minimization with J1036 is shown as a white ribbon. Residues in the active site are shown in stick representation and colored respectively.

**Table S1.** Pre-filtered Interbioscreen database (80 compounds) considered for the docking study.

| Comp. ID | GLIDE SP Score | Classical Hydroxamate –C=O-NH-OH | PAINS | MW | R/S | SMILES |
|---|---|---|---|---|---|---|
| **STOCK1S-00970** | -5.89 | x | | 297.29 | | O=C1N(CCCC(=O)N[O-])C(=O)c2c3c1cccc3ccc2 |
| **STOCK1S-05147** | -8.73 | | | 173.24 | | O=C(N([O-])C(C)C)NC(C)(C)C |
| **STOCK1S-05630** | -5.42 | x | | 292.40 | | Oc1c(cc(cc1C(C)(C)C)CCC(=O)N[O-])C(C)(C)C |
| **STOCK1S-13592** | -6.78 | | | 199.62 | | Clc1cc(NC(=O)N([O-])C)ccc1 |
| **STOCK1S-14427** | -6.82 | | | 286.72 | | Clc1ccc(NC(ON(CC)C(OCC)=O)=O)cc1 |
| **STOCK1S-44534** | -5.20 | x | | 150.58 | | ClCC(C(=O)N[O-])(C)C |
| **STOCK1S-44885** | -4.84 | | | 328.35 | | O(CCNC(=O)Nc1ccccc1)C(=O)N([O-])c1ccc(cc1)C |
| **STOCK1S-61698** | -7.24 | x | | 138.11 | | O=C(N[O-])c1nccnc1 |
| **STOCK1S-93629** | -4.33 | x | | 218.19 | | O=C(N[O-])c1[n+]([O-])c(nc2c1cccc2)C |
| **STOCK2S-07694** | - | x | | 235.05 | | Clc1cc(Cl)ccc1OCC(=O)N[O-] |
| **STOCK2S-62642** | -4.67 | x | | 226.66 | | Clc1c2c(sc1C(=O)N[O-])cccc2 |
| **STOCK2S-83315** | -7.01 | | x | 243.20 | | o1cccc1C1=[N+]([O-])c2c(N([O-])C1=O)cccc2 |
| **STOCK2S-84963** | -6.10 | x | | 235.05 | | Clc1cc(Cl)ccc1OCC(=O)N[O-] |
| **STOCK2S-87369** | -5.34 | x | | 263.10 | | Clc1cc(Cl)ccc1OCCCC(=O)N[O-] |
| **STOCK2S-99347** | - | | x | 287.68 | | Clc1ccc(cc1)C1=[N+]([O-])c2c(N([O-])C1=O)cccc2 |
| **STOCK3S-07818** | -5.06 | x | | 242.68 | | Clc1cc(C)c(OCCCC(=O)N[O-])cc1 |
| **STOCK3S-93087** | - | | | 317.33 | | O=C1N([O-])C(n2nc(cc2C)-c2ccccc2)=Nc2c1cccc2 |
| **STOCK3S-95273** | -7.53 | | | 295.28 | | Oc1cc(ccc1)\C=N/NC1=Nc2c(cccc2)C(=O)N1[O-] |
| **STOCK4S-02282**[*] | -3.60 | | | 351.81 | S | Clc1ccc(N2C(=O)[C@@H](N([O-])C(=O)CCCCCC)CC2=O)cc1 |
| **STOCK4S-02282**[*] | -3.92 | | | 351.81 | R | Clc1ccc(N2C(=O)[C@H](N([O-])C(=O)CCCCCC)CC2=O)cc1 |
| **STOCK4S-03815**[*] | -4.31 | | | 331.39 | S | O=C1N(Cc2ccccc2)C(=O)C[C@@H]1N([O-])C(=O)CCCCCC |
| **STOCK4S-03815**[*] | -3.47 | | | 331.39 | R | O=C1N(Cc2ccccc2)C(=O)C[C@H]1N([O-])C(=O)CCCCCC |
| **STOCK4S-11028**[*] | -3.81 | | | 335.35 | R | Fc1ccc(N2C(=O)[C@H](N([O-])C(=O)CCCCCC)CC2=O)cc1 |
| **STOCK4S-11028**[*] | -4.47 | | | 335.35 | S | Fc1ccc(N2C(=O)[C@@H](N([O-])C(=O)CCCCCC)CC2=O)cc1 |
| **STOCK4S-11661**[*] | -3.82 | | | 396.26 | S | Brc1ccc(N2C(=O)[C@@H](N([O-])C(=O)CCCCCC)CC2=O)cc1 |
| **STOCK4S-11661**[*] | -3.82 | | | 396.26 | R | Brc1ccc(N2C(=O)[C@H](N([O-])C(=O)CCCCCC)CC2=O)cc1 |
| **STOCK4S-25147** | -5.64 | | | 194.23 | R | S1CC(=O)N([O-])[C@H]1c1ccccc1 |
| **STOCK4S-25147** | -6.95 | | | 194.23 | S | S1CC(=O)N([O-])[C@@H]1c1ccccc1 |
| **STOCK4S-27444**[*] | -3.73 | | | 317.36 | R | O=C1N(C(=O)C[C@H]1N([O-])C(=O)CCCCCC)c1ccccc1 |
| **STOCK4S-27444**[*] | -2.94 | | | 317.36 | S | O=C1N(C(=O)C[C@@H]1N([O-])C(=O)CCCCCC)c1ccccc1 |
| **STOCK4S-31959**[*] | -5.13 | | | 331.39 | S | O=C1N(C(=O)C[C@@H]1N([O-])C(=O)CCCCCC)c1ccc(cc1)C |
| **STOCK4S-31959**[*] | -4.49 | | | 331.39 | R | O=C1N(C(=O)C[C@H]1N([O-])C(=O)CCCCCC)c1ccc(cc1)C |
| **STOCK4S-48892**[*] | -5.64 | | | 289.31 | S | O=C1N(C(=O)C[C@@H]1N([O-])C(=O)CCCC)c1ccccc1 |
| **STOCK4S-48892**[*] | -6.60 | | | 289.31 | R | O=C1N(C(=O)C[C@H]1N([O-])C(=O)CCCC)c1ccccc1 |
| **STOCK4S-53643**[*] | -5.13 | | | 337.78 | S | Clc1ccc(N2C(=O)[C@@H](N([O-])C(=O)CCCCC)CC2=O)cc1 |
| **STOCK4S-53643**[*] | -4.47 | | | 337.78 | R | Clc1ccc(N2C(=O)[C@H](N([O-])C(=O)CCCCC)CC2=O)cc1 |
| **STOCK4S-57300** | -5.27 | x | | 232.22 | R | O(C)c1ccc(cc1)-c1[nH]nc(c1)C(=O)N[O-] |
| **STOCK4S-57300** | -6.63 | x | | 232.22 | S | O(C)c1ccc(cc1)-c1n[nH]c(c1)C(=O)N[O-] |
| **STOCK4S-73929** | -3.08 | | | 343.75 | R | Clc1ccc(N2C(=O)[C@@H](N([O-])C(=O)c3ccccc3)CC2=O)cc1 |
| **STOCK4S-73929** | -3.30 | | | 343.75 | S | Clc1ccc(N2C(=O)[C@H](N([O-])C(=O)c3ccccc3)CC2=O)cc1 |
| **STOCK4S-78560**[*] | -3.46 | | | 303.34 | S | O=C1N(C(=O)C[C@@H]1N([O-])C(=O)CCCCC)c1ccccc1 |
| **STOCK4S-78560**[*] | -5.99 | | | 303.34 | R | O=C1N(C(=O)C[C@H]1N([O-])C(=O)CCCCC)c1ccccc1 |
| **STOCK4S-84022** | - | | | 255.26 | | O=C1N([O-])C(n2nc(cc2C)C)=Nc2c1cccc2 |
| **STOCK5S-04928** | -6.87 | | | 193.21 | | S=C1Nc2c(cccc2)C(=O)N1[O-] |
| **STOCK5S-12121** | - | | | 372.20 | | Brc1ccc(cc1)/C(=N/NC1=Nc2c(cccc2)C(=O)N1[O-])/C |
| **STOCK5S-21668** | - | | | 358.17 | | Brc1ccc(cc1)\C=N\NC1=Nc2c(cccc2)C(=O)N1[O-] |
| **STOCK5S-30259** | -5.46 | | | 283.27 | | o1cccc1/C(=N\NC1=Nc2c(cccc2)C(=O)N1[O-])/C |
| **STOCK5S-30523** | -7.06 | | x | 295.28 | | Oc1ccc(cc1)\C=N/NC1=Nc2c(cccc2)C(=O)N1[O-] |
| **STOCK5S-31605** | -6.23 | | | 339.33 | | O(C)c1cc(ccc1OC)\C=N/NC1=Nc2c(cccc2)C(=O)N1[O-] |
| **STOCK5S-33544** | - | | x | 345.34 | | Oc1ccc2c(cccc2)c1\C=N\NC1=Nc2c(cccc2)C(=O)N1[O-] |

| | | | | | | |
|---|---|---|---|---|---|---|
| **STOCK5S-36950** | - | | | 313.72 | | Clc1ccc(cc1)\C=N\NC1=Nc2c(cccc2)C(=O)N1[O-] |
| **STOCK5S-38273** | - | | | 293.31 | | O=C1N([O-])C(=Nc2c1cccc2)N\N=C(/C)\c1ccccc1 |
| **STOCK5S-40226** | -2.39 | | | 295.27 | R | O=C(Nc1cc([N+](=O)[O-])ccc1)N([O-])[C@@H](C\C(=N/O)\C)C |
| **STOCK5S-40226** | -2.80 | | | 295.27 | S | O=C(Nc1cc([N+](=O)[O-])ccc1)N([O-])[C@H](C\C(=N/O)\C)C |
| **STOCK5S-46144** | -7.64 | | x | 323.33 | | Oc1ccc(cc1)/C(=N\NC1=Nc2c(cccc2)C(=O)N1[O-])/CC |
| **STOCK5S-46331** | -8.10 | | x | 309.30 | | Oc1ccc(cc1)/C(=N\NC1=Nc2c(cccc2)C(=O)N1[O-])/C |
| **STOCK5S-46688** | -4.48 | | | 284.72 | | Clc1ccc(NC(=O)N([O-])C(\C(=N/O)\C)(C)C)cc1 |
| **STOCK5S-50748** | - | | | 293.31 | | O=C1N([O-])C(=Nc2c1cccc2)N\N=C\c1ccccc1C |
| **STOCK5S-52688** | - | | | 327.75 | | Clc1ccc(cc1)/C(=N/NC1=Nc2c(cccc2)C(=O)N1[O-])/C |
| **STOCK5S-54734** | - | | | 313.72 | | Clc1ccccc1\C=N\NC1=Nc2c(cccc2)C(=O)N1[O-] |
| **STOCK5S-56064** | - | | | 307.33 | | O=C1N([O-])C(=Nc2c1cccc2)N\N=C\c1cc(ccc1C)C |
| **STOCK5S-58555** | -3.79 | | x | 295.28 | | Oc1ccccc1\C=N/NC1=Nc2c(cccc2)C(=O)N1[O-] |
| **STOCK5S-73318** | - | | | 285.33 | | O=C1N([O-])C(=Nc2c1cccc2)N\N=C/1\CCCCCC\1 |
| **STOCK5S-81005** | -5.18 | | | 280.27 | | O=C1N([O-])C(=Nc2c1cccc2)N\N=C/c1ccncc1 |
| **STOCK6S-00835** | -5.95 | | | 273.13 | R | Brc1ccc(cc1)[C@H]1SCC(=O)N1[O-] |
| **STOCK6S-00835** | -6.69 | | | 273.13 | S | Brc1ccc(cc1)[C@@H]1SCC(=O)N1[O-] |
| **STOCK6S-33232** | -6.95 | | | 330.36 | | S1C[C@@H](N(C1)C(OC(C)(C)C)=O)C(ON1C(=O)CCC1=O)=O |
| **STOCK6S-33232** | -4.87 | | | 330.36 | | S1C[C@H](N(C1)C(OC(C)(C)C)=O)C(ON1C(=O)CCC1=O)=O |
| **STOCK6S-38389** | -5.85 | | | 295.29 | | O=C1N(OC(=O)c2ccc(cc2)-c2ccccc2)C(=O)CC1 |
| **STOCK7S-02226** | -5.64 | | | 246.21 | | O=C1N([O-])C=CC=2n3nc(nc3N=CC1=2)COC |
| **STOCK7S-02759** | -5.83 | | | 201.16 | | O=C1N([O-])C=CC=2n3nccc3N=CC1=2 |
| **STOCK7S-16492** | -5.83 | x | | 161.53 | | Clc1[nH]nc(n1)C(=O)N[O-] |
| **STOCK7S-16492** | -5.19 | x | | 161.53 | | Clc1[nH]c(nn1)C(=O)N[O-] |
| **STOCK7S-16696** | -6.48 | | | 235.22 | | O(C)c1cc2c(N=C(N([O-])C2=O)C)cc1OC |
| **STOCK7S-26244** | -5.70 | | | 202.15 | | O=C1N([O-])C=CC=2n3nccc3N=NC1=2 |
| **STOCK7S-43289** | -7.59 | | | 142.11 | | O1N(C(=O)C)C(=O)C=C1N |
| **STOCK7S-43543** | -6.50 | | | 204.19 | | O1N(C(=O)c2ccccc2)C(=O)C=C1N |
| **STOCK7S-45626** | -7.73 | | | 170.17 | | O1N(C(=O)C(C)C)C(=O)C=C1N |
| **STOCK7S-47746** | -5.38 | | | 184.19 | | O1N(C(=O)CC(C)C)C(=O)C=C1N |
| **STOCK7S-49196** | -4.56 | | | 256.62 | | Clc1cc(ccc1F)C(=O)N1OC(N)=CC1=O |
| **STOCK7S-49604** | -7.96 | | | 220.02 | | BrC=1C(=O)N(OC=1C)C(=O)C |
| **STOCK7S-50104** | -5.85 | | | 258.16 | | Fc1c(F)c(F)ccc1C(=O)N1OC(N)=CC1=O |
| **STOCK7S-50249** | -4.71 | | | 172.14 | | O1N(C(OCC)=O)C(=O)C=C1N |
| **STOCK7S-51469** | -6.07 | | | 210.23 | | O1N(C(=O)C2CCCCC2)C(=O)C=C1N |
| **STOCK7S-52645** | -7.04 | | | 282.09 | | BrC=1C(=O)N(OC=1C)C(=O)c1ccccc1 |
| **STOCK7S-53377** | -7.17 | | | 274.06 | | Clc1nc(Cl)ccc1C(=O)N1OC(N)=CC1=O |
| **STOCK7S-53722** | -6.24 | | | 283.63 | | Clc1ccc([N+](=O)[O-])cc1C(=O)N1OC(N)=CC1=O |
| **STOCK7S-54511** | -7.05 | | | 240.16 | | Fc1cccc(F)c1C(=O)N1OC(N)=CC1=O |
| **STOCK7S-54658** | -7.14 | | | 240.16 | | Fc1ccc(F)cc1C(=O)N1OC(N)=CC1=O |
| **STOCK7S-54794** | -7.62 | | | 288.18 | | FC(F)(F)Oc1ccc(cc1)C(=O)N1OC(N)=CC1=O |
| **STOCK7S-55112** | -6.18 | | | 273.07 | | Clc1cc(cc(Cl)c1)C(=O)N1OC(N)=CC1=O |
| **STOCK7S-55612** | -6.84 | | | 239.62 | | Clc1ncc(cc1)C(=O)N1OC(N)=CC1=O |
| **STOCK7S-56364** | -6.61 | | | 284.07 | | Brc1cc(cnc1)C(=O)N1OC(N)=CC1=O |
| **STOCK7S-59574** | - | | | 312.37 | | O=C1N(OC)C(n2nc(cc2CC(C)C)C)=Nc2c1cccc2 |
| **STOCK7S-64837** | - | | | 297.34 | | O=C1N([O-])C(n2nc(cc2CC(C)C)C)=Nc2c1cccc2 |
| **STOCK7S-69412** | - | | | 316.13 | | Brc1ccccc1C1=Nc2c(cccc2)C(=O)N1[O-] |

*Compounds that were selected for biological testing (highlighted in green); - = Compounds for which no docking pose was generated since they failed to correctly coordinate to the zinc ion; MW = Molecular weight; For chiral compounds both R/S stereoisomers were considered.

**Table S2.** smHDAC8/inhibitor X-ray structure. Data collection and refinement statistics.

| **Data collection** | **smHDAC8-J1036** |
|---|---|
| Space group | P1 |
| **Cell dimensions** | |
| a, b, c (Å) | 70.71 70.69 99.19 |
| α, β, γ (°) | 75.33 77.78 84.8 |
| Resolution (Å)* | 41.21 - 1.55 (1.59 - 1.55) |
| Rmerge (%) | 6.3 (74.7) |
| I / σI | 11.87 (1.94) |
| Completeness (%) | 95.6 (87.8) |
| Redundancy | 3.5 (2.9) |
| CC(1/2) | 99.4 (60) |
| **Refinement** | |
| Resolution (Å) | 41.96-1.55 |
| No. reflections | 253034 |
| Rwork / Rfree (%) | 19.94/22.27 |
| **No. atoms** | |
| Protein | 1628 |
| Ligand/ion | 90 |
| Water | 599 |
| **B-factors** | |
| Protein | 29.01 |
| Ligand/ion | 43.84 |
| Water | 34.93 |
| **R.m.s deviations** | |
| Bond lengths (Å) | 0.007 |
| Bond angles (°) | 0.86 |

*Values in parentheses are for highest-resolution shell.
